# 

**Published:** 1895-06-08

**Authors:** 


### CONTENTS.

**The Modern Treatment of Heart Disease** - - - - 159
**Some Aspects of Research in Medicine** - - - - 160
**The Classification of Aneurisms.** By A. PEARCE GOULD, F.R.C.S. - - - - 161
**Genius and Insanity** - - - - 162
**Medical Progress and Hospital Clinics—**
Clinical Aspects of Scoliosis - - - - 165
Internal Antiseptics - - - - 166
PROGRESS IN MEDICINE.—Diseases of Children - - - - 168
PROGRESS IN SURGERY.—The Surgery of the Spleen and Pancreas 169
NEW APPLIANCES AND THINGS MEDICAL - - - - 170
**Annotations.**—Should Measles be Notified?—Alcohol in Workhouses—Our Hospital Sunday Supplement - - - - 163
**Notes and News** - - - - 164
**The Institutional Workshop:—**
HOSPITAL CONSTRUCTION - - - - 171
THE STORY OF THE INSANE FROM YEAR TO YEAR - - - - 172
PRACTICAL DEPARTMENTS.—Floors for Hospital Wards - - - 173
HOSPITAL FESTIVALS AND MEETINGS - - - - 173
CONSTRUCTION NOTES - - - - 174
**Editor's Letter Box** - - - - 174
**Scraps and Gleanings** - - - - 174

### EXTRA SUPPLEMENT.—THE NURSING MIRROR.

**News from the Nursing World.**—The Hospital for Sick Children—"The Hospital" Convalescent Fund—Freidenheim—Matrons of Cottage Hospitals—Practical Orders—Economy *versus* Efficiency—Endangered Lives—A Legacy for Bucks—Mentally and Bodily Affliction—Progress in Rome—Short Items - - - - lxv

**Elementary Anatomy and Surgery for Nurses.** By W. McADAM ECCLES, M.D., M.S., F.R.C.S.—XX.—Hæmorrhage from Particular Regions—Aneurism—Nævus - - - lxvii

**The Society of Trained Masseuses** - - - - lxvii

**Appointments** - - - - lxvii

**Boston Training School for Nurses** - - - - lxviii

**The Indian Hospital, British Columbia** - - - - lxviii

**Everybody's Opinion** - - - - lxix

**Royal National Pension Fund for Nurses** - - - - lxix

**Notes and Queries** - - - - lxix

**Nursing in Military Hospitals.**—Time-expired Men - - lxx

**Asylum News and Nursing** - - - - lxx

**A New Hospital for Crewe** - - - - lxx

**The Congress at Geneva** - - - - lxx

